# *In vitro* corrosion studies of stainless-steel dental substrates during *Porphyromonas gingivalis* biofilm growth in artificial saliva solutions: providing insights into the role of resident oral bacterium†

**DOI:** 10.1039/d0ra05500j

**Published:** 2020-08-24

**Authors:** Ubong Eduok, Jerzy Szpunar

**Affiliations:** Department of Mechanical Engineering, College of Engineering, University of Saskatchewan 57 Campus Drive Saskatoon S7N 5A9 Saskatchewan Canada ubong.eduok@usask.ca +1 (306) 966 5427 +1 (306) 966 7752

## Abstract

Stainless-steel AISI 321 is an effective material for fabricating dental crowns and other implants utilized dental restorative protocols for elderly and pediatric populations. This unique clinical application is possible through the mechanical stability and corrosion-resistance properties of this metallic material. However, stainless-steel dental implants eventually fail, leading to the creation of surface cavities and cracks within their microstructures during persistent mechanical stresses and biocorrosion. In this study, the *in vitro* corrosion behaviour of a medical-grade stainless-steel dental substrate was investigated during *Porphyromonas gingivalis* biofilm growth process in artificial saliva culture suspension (ASCS). Among the causative bioagents of corrosion, *P. gingivalis* was chosen for this study since it is also responsible for oral periodontitis and a major contributing factor to corrosion in most dental implants. Increased *P. gingivalis* growth was observed within the incubation period under study as compact cellular clusters fouled the metal surfaces in ASCS media. This led to the corrosion of steel substrates after bacterial growth maturity within 90 days. Corrosion rate increased with higher CFU and bacterial incubation period for all test substrates due to biocorrosion incited by the volatile sulphide products of *P. gingivalis* metabolism. The presence of some of these volatile compounds has been observed from experimental evidences. Significant anodic degradation in the forms of localized pitting were also recorded by surface analytical techniques. Residual fluorinated ions within the ASCS media also increased the rate of anodic dissolution due to media acidity. This study has provided extensive insights into the fate of stainless-steel dental crown in oral environments infected by a resident oral bacterium. Influences of oral conditions similar to fluoride-enriched mouthwashes were reflected in a view to understanding the corrosion patterns of stainless-steel dental substrates.

## Abstract 2

A stainless-steel 321 dental substrate significantly corroded within *Porphyromonas gingivalis* growth culture in artificial saliva culture suspension, with and without NaF additive.

## Graphical abstract

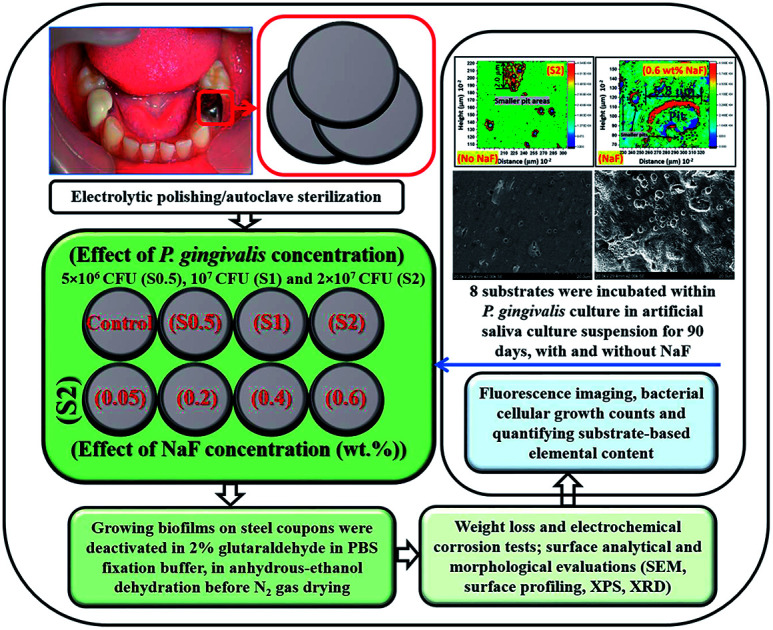

## 1. Introduction

Corrosion of iron is a combination of dual and rather complex oxidation–reduction processes involving cascades of electron releases. Its oxidation half-reaction dominates the anodic process in acidic solutions, and in some cases, the better understanding of the net corrosion mechanism and associated kinetics may be determined by iron oxidation.^1^ The rate of iron dissolution in this medium may depend mostly on its metallic content (*e.g.* the presence of alloying elements in stainless steel) and its electrochemical behavior at defined potentials as well as pH values. Iron dissolution within the corrosive acid solution (eqn (1)) may also be accompanied by cathodic oxygen reduction and/or hydrogen evolution reaction (eqn (2) and (3)), depending on the media pH.^2^ At passive regions, the formation of a protective oxide films delays corrosion since the rate of passivation exceeds corrosion; this is common in stainless steel metal substrates.^1,2^

1 Fe → Fe^2+^ + 2e^−^

2 O_2_ + 4H^+^ + 4e^−^ → 2H_2_O

3 O_2_ + 2H_2_O + 4e^−^ → 4H^+^

**Fig. 1:**
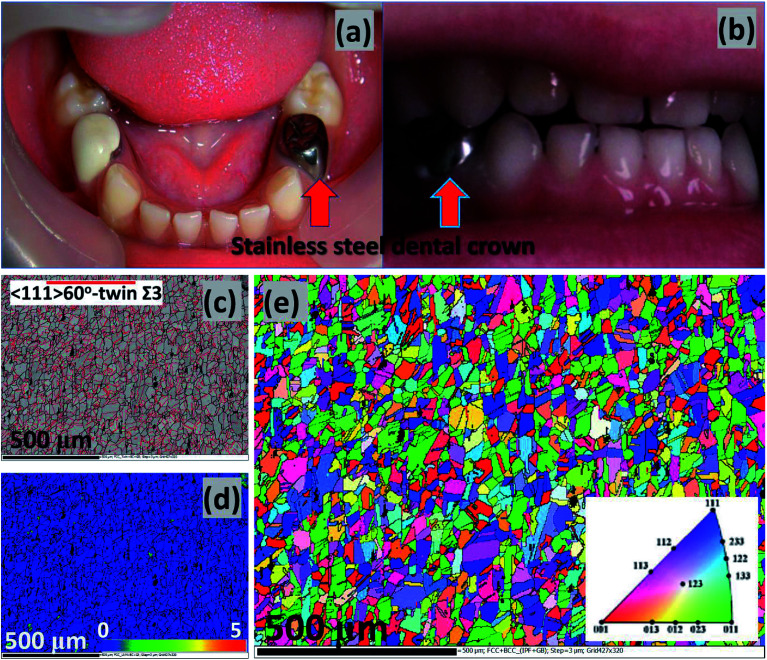
Typical stainless-steel crowns after cementation; authors in this study^11^ were investigating the effects of microbial adhesion of preveneered and stainless-steel crowns (a). The intercuspation image from a patient's post-treatment (b) procedure on a bite wound treated according to the Hall technique; the stainless-steels crowns cover the premolars.^12^ Collective EBSD maps of the stainless-steel similar to those utilized within this study: (c) band contrast and twin, (d) KAM and (e) EBSD orientation/inverse pole figure (IPF);^41^ images are reproduced with permission. Apart from titanium, niobium and vanadium are also strong carbon-form elements incorporated with AISI 321 stainless-steel dental substrates to prevent depleting chromium as chromium carbides precipitates from around the grain boundaries, in turn, inhibiting intergranular corrosion and intergranular corrosion stress corrosion cracking (a, b and c–e are reproduced with permissions from ref. 11,^12^ and 41, respectively).

Corrosion is a huge problem in dental hygiene and maintenance of the mechanical integrity of metallic implants. The use of stainless-steel metallic crowns in clinical restorative protocols has been popular in dental care since the 1950's. This is could be heavily linked with the invaluable stainless-steels component materials utilized in fabrication of dental tools for degrading primary teeth.^3^ The unique mechanical properties of stainless steels coupled with their heat and corrosion-resistance and their low maintenance have made them durable and superior crown materials over amalgam and other common dental crown restorative materials.^4^ Independent of the global widespread of stainless-steel crown usage, there are also some recorded quality failures,^5^*e.g.* in the forms of defective margins,^6,7^ and when there are compromises in oral health due to gingivitis.^8,9^ However, the success rate for stainless-steel crowns far exceeds those of Class II amalgam fillers, and their failure rates are also lower than those of amalgam restorative materials (*e.g.* Cu, Hg, Ag and Sn).^10^ The dental stainless-steel crowns may be needed to protect and restore weak teeth and implants, cover installed fillings, support dental bridges in place, *etc.* Typical stainless-steel crowns utilized in restorative dental care are presented in Fig. 1(a) and (b);^11,12^ both permanent and temporary crowns could be made from stainless-steel alloys such as AISI 305, 316, 321, *etc.*^13^

Stainless-steel dental implants have also been an effective option for lost teeth restoration,^14^ and recorded clinical successes are linked with their mechanical integrity (*e.g.* fatigue and tensile strengths, corrosion and fracture resistances).^15–17^ Despite their remarkable properties, these implants still eventually fail and those removed from oral cavities bear surface cracks within their microstructures due to persistent mechanical stresses and microbiologically induced corrosion (MIC). The prevalence of periodontitis may be another contributing source identifier for implant failure.^18,19^ Rodrigues *et al.*^20^ have recorded evidence of surface pitting on retrieved dental implants. Implant failures are the consequences of inherent metallic corrosion and are also a direct contribution of some resident oral bacteria within the gum lines. Among the causative bioagents of corrosion, *Porphyromonas gingivalis* (*P. gingivalis*), a periodontitis pathogen, has become a key factor in the corrosion of most dental implants.^21^ Like most oral bacteria, *P. gingivalis* initiates corrosion in a mechanism linked with the biological activities within its biofilm and inherent metabolic product releases, notably, volatile sulfur compounds.^22^ These volatile compounds (*e.g.* hydrogen sulfide (H_2_S), methyl mercaptan (CH_3_SH) and dimethyl sulfide ((CH_3_)_2_S)) alter metallic microstructures and may also contribute to tensile stresses and/or sulfide stress corrosion cracking. Their presence impacts adversely on material integrity of metals while also contributing to their corrosion by reducing the pH of their environments.^23–25^ In patients suffering from periodontal diseases, metallic release during corrosion may change the pH around dental tissues while inflammatory tissue ailments involving peri-implantitis due to oral bacterial infections may also lead to loss of metallic implant materials, fatigue and subsequently, fractures.^26,27^ These biofilm-led biological processes contribute to the creation of corrosive concentration cells on these metallic surfaces, thereby increasing the kinetics of anodic reactions, hence, accelerating the rate of corrosion. This *P. gingivalis* bacterial pathogen has led to the sufferings of 47% US periodontitis adult patients and 538 infected people worldwide (of which 276 have lost their teeth already).^28^*P. gingivalis* is a biofilm forming bacterium, especially on solid surfaces (*e.g.* teeth surfaces and those of dental implants). Since *P. gingivalis* is a common oral bacterium^29^ whose counts would normally increase during peri-implantitis,^30^ there is a need to investigate its growth effects on medical-grade stainless steel used in fabricating dental crown implants.

Stainless steel is commonly used as dental crown and other forms of implants within oral environments with varying temperatures and pHs. In oral fluids, these steel-based dental crown attachments have the potential to corrode due to the presence of inherent biological and chemical agents. Corrosion may proceed, leading to continuous degradation during electrochemical processes leading to corrosion attacks within hostile electrolytic oral environments close to the teeth.^31^ A number of authors have studied the corrosion of stainless steel in the presence of oral biofilm-forming bacteria in dentistry.^32–34^ Laurent *et al.*^32^ have investigated the degradation of two dental alloys, Ni–Cr and Au-based alloys, in the presence of *Actinomyces viscosus* cultured in modified Fusayama artificial saliva. Authors observed an absence of oxygen contributed to increase in polarization resistance of the Ni–Cr. However, they also observed a steady decrease for the Au alloy due to inherent metabolites released by bacterium within the culture media. Another research group^33^ also studied the same effect on stainless steel using *Lactobacillus* and *Leuconostoc* lactic acid bacteria (LAB) isolates. In all, authors observed these LAB strains inhibited *L. monocytogenes* biofilm formation and growth on the metallic substrate hence altering its corrosion rate. A similar trend was also observed for lettuce and on a MBEC Assay®'s Biofilm Inoculator. The extent corrosion of orthodontic archwires in artificial saliva culturing *Lactobacillus reuteri* has also been investigated by Trolic *et al.*^34^ The bacterial growth on the metallic substrate influenced the corrosion rate by contributing to surface pitting. Fatani *et al.*^35^ have reported an *in vitro* assessment of the antibacterial properties of Ag/TiO_2_ coated stainless-steel orthodontic brackets against *P. gingivalis* and *Streptococcus mutans* growths. Authors observed that the TiO_2_–Ag film on the steel brackets prevented against bacterial surface adhesion and biofilm formation due to its antibacterial properties and resistance toward plaque accumulation. Papaioannou *et al.*^36^ also studied the corrosion of orthodontic stainless-steel brackets in *P. gingivalis* culture medium incubated at 37 °C for 3 days under anaerobic conditions in saliva solutions of healthy adults. They observed that the salivary pellicle facilitated the adhesion and formation of *P. gingivalis* biofilms on orthodontic brackets.

The biocorrosion effects of a specific oral bacterium, *P. gingivalis*, on titanium surfaces have also been extensively reported.^29,37–39^ Corrosion related to titanium alloys has effects on the health of peri-implant tissues as well as long term survival of these metal dental implants. Inherent long-term and continuous corrosion leads to the release of ions into the peri-implant tissues and subsequent disintegration of the implants. These events contribute to material fatigue and fractures.^40^ The effects of corrosion on titanium alloys must have been extensively investigated, however, there are only a few reports on medical-grade stainless-steel dental substrates involving the influences of its metabolic products on corrosion within any know oral medium.^35,36^ This is the first featured study designed to determine the influence of *P. gingivalis*'s metabolic products with its culture medium on the corrosion behaviour of stainless steel. Beyond corrosion electrochemistry, the composition of adhering bacterial biofilms on steel is investigated in a view to establishing its contribution to reduced corrosion resistance of this dental substrate in artificial saliva with NaF. Corrosion investigations in acidic oral conditions containing fluoride ions was necessary in order to depict fluoride-containing oral environments during mouthwash and when the mouth is gargle with toothpastes and prophylactic gels to avoid dental cavity and surface sensitivity. These dental products are also known to reduce oral pH,^31^ and the accelerated corrosion-causing effects on their halide ions components on a typical stainless-steel dental substrate is investigated within this study.

## 2. Materials and methods

### 2.1. Reagents and chemicals

Sulfuric acid (99.999%) and phosphoric acid (85 wt% in H_2_O) were utilized in metal polishing and were purchased from Sigma Aldrich. Hemin (from porcine, ≥97%), 2-methyl-1,4-naphthoquinone sodium bisulfite (≥95%), l-cysteine hydrochloride monohydrate (≥98%) and yeast were also purchased from Sigma Aldrich and added to the bacterial culture medium, BHI (Brain Heart Infusion) broth. Hydrochloric acid (ACS reagent, 37%) and sodium fluoride (NaF, ≥99%) were also purchased from the same outlet. The chemicals used in preparing the artificial saliva medium for this study are Sigma Aldrich products and are presented in Table 1.

**Table 1:** Chemical composition of artificial saliva utilized within this study (*except otherwise stated, contents were measured in mM); with slight variation from those reported in ref. 43

Major constituents	Contents	Concentration (mM*)
Salts	Potassium chloride	15.6
	Monopotassium phosphate	2.6
	Sodium dihydrogen phosphate	2.6
	Sodium chloride	16.0
	Calcium chloride	1.4
Basal amino acids	Alanine	0.04
	Cysteine	0.05
	Glycine	0.119
	Leucine	0.022
	Lysine	0.019
	Phenylalanine	0.018
	Tyrosine	0.012
	Valine	0.016
Vitamins	Ascorbic acid	0.01
	Thiamine	0.00002
	Riboflavin	0.00013
Other additives	Albumin	0.0004
	Mucin	8 g L^−1^
	Urea	2.9

### 2.2. Preparation procedure for steel substrate

An AISI 321 austenitic stainless steel was utilized as the metallic dental substrate in this study. It was used as received without further deformation with its initial microstructure similar to those presented in Fig. 1(c)–(e). The chemical composition (wt%) of this metallic substrate is presented in Table 2. Smaller round-disk shaped (with 1 cm diameter and 1 cm height) coupons were cut out of a bigger metallic mass. These coupons were not made into the engineered-shaped dental crowns in order to allow for sufficient unimpacted surface area for corrosion test while also avoiding premature corrosion on stressed edges. These coupons were then electrolytically polished using a suspension consisting of 35% H_2_SO_4_, 45% H_3_PO_4_ and 20% de-ionized water, degreased in 50 : 50 acetone/ethanol suspension by ultrasonication (Branson M1800 Ultrasonic Cleaner) before drying them in pure liquid N_2_.^41^ The final cleaning step involved sterilization using an autoclave prior to corrosion test in bacterial culture. Only the metallic coupons used for the electrochemical tests were sealed in epoxy materials. The microstructure of this metal is similar to those recorded using an electron backscatter diffraction (EBSD) technique in ref. 41. Its microstructural maps reveal the formation of annealing twin boundaries (a), reduced magnitude of Kernel average misorientation (KAM), hence low strain (b), as well as uneven and rather randomized orientations (c). KAM values are a measure of misorientation of localized grains in terms of strain distribution; they also quantify these misorientations as averages from measured reference points with respect to their respective neighboring points.

**Table 2:** Chemical composition (wt%) of stainless steel utilized as dental substrate material within this study

Cr	Ni	Mn	Mo	Si	Ti	Cu	Co	C	Fe
17.61	9.17	1.56	0.42	0.40	0.36	0.30	0.15	0.044	Balance

### 2.3. Bacterial growth and cultivation conditions

**Fig. 2:**
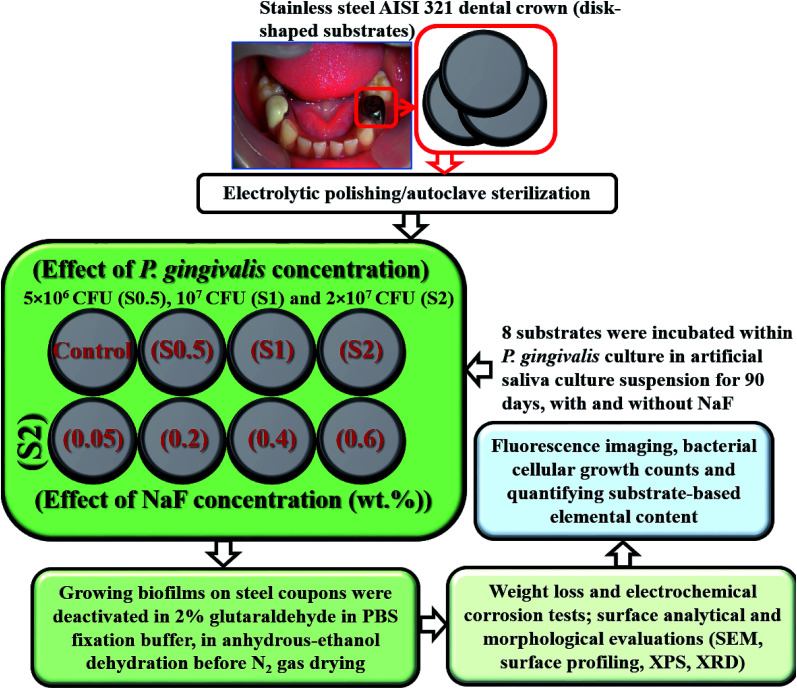
A flow chart showing the study design, type and number of test samples utilized in the present study.

*P. gingivalis* ATCC 33277 was used as the test bacterium in this biocorrosion study. It was cultured in an anaerobic chamber with a nutrient-rich BHI broth with the following components per litre (L) of sterile water: 5 g hemin, 5 g 2-methyl-1,4-naphthoquinone sodium bisulfite, 1 g l-cysteine hydrochloride monohydrate and 10 g yeast supplements.^29^ This chamber was first sparged with pure N_2_ gas to remove dissolved oxygen before the culturing; the pH of the culture-media content was maintained at 7 (S220-B Mettler Toledo). Prior to bacterial culture, *P. gingivalis* cells were preactivated with their culture plate at 38 °C for 18 h within an incubator. After the culture duration, the bacterial cells were separated from the medium by ultracentrifugation (10 000 rpm, 5 min, Beckman Ultracentrifuge) at 6 °C. The separated cellular suspension was then diluted to a 0.5 optical density at 600 nm (Agilent 8453 UV-visible spectrophotometer). The corresponding cellular concentration was then computed before quantitatively transferring to 10 mL BHI culture medium.^42^ After this preculture, individual sterile stainless-steel disk-shaped coupons were then completely immersed in 1 L artificial salivary culture suspension (ASCS)^43^ in Culture Multiwell Plates (Sigma Aldrich), capped inside sealed small glass chambers utilized as lab-made bioreactors, and further sealed in transparent bags before placing them in an incubated anaerobic chamber at 38 °C for defined culture duration. Each well was inoculated with 10^7^ cells per mL of viable 3 day old inoculum of *P. gingivalis* at complete 100% deoxygenated medium at pH 7. The needed electrochemical corrosion tests were conducted as well as the SEM imaging of adhering biofilms on the surface of the stainless-steel coupons. Prior to these tests, the biofilms were deactivated by placing all retrieved coupons in 2% glutaraldehyde in PBS fixation buffer followed by anhydrous-ethanol dehydration and N_2_ gas drying.^42^ This later rinsing procedures were conducted on each dental substrate, one at a time. The flow chart showing the study design, type and number of test samples utilized in the present study are presented in Fig. 2.

### 2.4. Analyses of colonized bacterial biofilms on stainless steel

In order to view the morphologies of adhering *P. gingivalis* biofilms on the metal surfaces in line with bacterial growth, these dental substrate coupons were sequentially treated to a fixation buffer for 3 h. They were then dehydrated in ethanolic solutions stepwisely, from 50 to 100% at 20 min each step before freeze-drying them with liquid N_2_. After thin-layer Au coating on each metallic coupon, the adhering bacterial biofilms as well as the after-corrosion changes (in the form of surface pits) were then imaged using scanning electron microscopy (SEM Hitachi SU6600 scanning electron microscope, Hitachi High-Tech.) at appropriate accelerating voltage. In cases were distinct surface images were difficult to observed due to overcrowding, these substrates were repeatedly washed using a solution of trypsin in sterile water for 12 h in order to further to remove some adhering *P. gingivalis* biofilms. The chemical composition of these biofilms was also determined using X-ray photoelectron spectroscopy XPS Kratos AXIS Supra system equipped with a 500 mm Rowland circle monochromated Al Kα (1486.6 eV) source and combined hemi-spherical and spherical-mirror analyzers. A spot size of hybrid slot (300 × 700 microns) was used. All survey scan spectra were collected in the 0–1200 binding energy range in 1 eV steps with a pass energy of 160 eV.^44^ High resolution scans of multiple regions were also conducted using 0.05 eV steps with a pass energy of 20 eV. An accelerating voltage of 15 keV and an emission current of 15 mA was used for analyses.^44^ Inherent pH changes within the bacterial culture media were also monitored periodically. The rates of evolution of volatile sulfur compounds in the presence of these substrates within the culture media were also determined using series of gas sampling pump kits and accessories (GV-100S, Gastec) coupled to detector tubes. The chemistry of adhering fluoride complex thin films on impacted stainless-steel substrates in the culture media containing varying concentrations of NaF was also carried out by means of X-ray diffractometry (Bruker D8 Discover XRD Diffractometer) at 40 mA and 40 kV with CuKα radiation. All spectra were recorded at 0.02 step size.

### 2.5. Biological staining and CLSM imaging

These metal-surface adhering bacterial biofilms were also imaged using confocal laser scanning microscopy (CLSM) but clearer images were collected using transparent microscope coverslips. Biofilm-bound and stained glass-slide substrates were incubated for 10 min in the dark and fixated using diluted glycerol/PBS solution before imaging with the aid of a Nikon TE2000-U fluorescence microscope. The *P. gingivalis* biofilm stained were exposed to the Filmtracer™ LIVE/DEAD® Biofilm Viability Kit (Thermo Fisher Scientific).^45^ This kit was used based on the efficiency of its two-color fluorescence assay for determining bacterial viability. For visual data collection, the bacterial cells were close enough to the surface, so there was no need to view stack images within the biofilm depth using the ZEN lite Digital Imaging Software.

### 2.6. Weight loss and electrochemical corrosion measurements

The rates of corrosion of stainless-steel coupons in the artificial salivary culture media (chemical composition are presented in Table 1) were determined by weight loss experiments using (eqn (4)).^42^ This was done after weighing (Sartorius, ±0.01 mg) individual metallic substrates, in triplicate, prior to and after the incubation period. The standard practice for preparing, cleaning, and evaluating corrosion test specimens, including all pre-weighing procedures were conducted in line with the protocol enlisted within ASTM G1-03 standard.^46^ Electrochemical measurements were also conducted on each substrate at different culture durations. These tests were conducted using a three-electrode cell system composing of a platinum auxiliary electrode and Ag/AgCl (sat. KCl) reference electrode (in a Luggin capillary) connected to Potentiostat/Galvanostat/ZRA (Interface 1000, Gamry Instruments). The corrosion-impacted stainless-steel dental substrates were utilized as working electrodes within artificial saliva at pH 6.5 at 38 °C. Tafel polarization experiment was conducted by applying from −0.5 up to 0.5 V *vs.* (Ag/AgCl (sat. KCl)) at a sweep rate of 0.5 mV s^−1^. Both anodic and cathodic polarization measurements were conducted in the same experimental run at the end of the 90 days. EIS measurements were measured at Open Circuit Potential (OCP) with respect to the reference electrode within 0.1 to 10 000 Hz frequency range. EIS spectra were recorded after applying an AC signal of 10 mV (rms) using the single sine technique. An equivalent circuit simulation program with EChem Analyst was utilized for all data analyses, creating of equivalent circuit models and fitting of experimental data. Before the theoretical data fitting to the circuit models, their linearity was determined using Kramers–Kronig transformation. The amounts of elements released from the bulk metal substrates as ions into the culture medium per surface area was also measured in ppm using inductively coupled plasma mass spectrometer (ICP-MS, Thermo Fisher Scientific). Before determining the concentrations of ions within these media, the test solutions were appropriately diluted with 1 M HCl in vortex mixer for 1 min.4
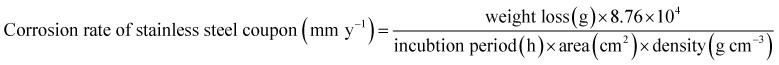


## 3. Results and discussion

### 3.1. Bacterial colonization and fluorescence imaging

**Fig. 3:**
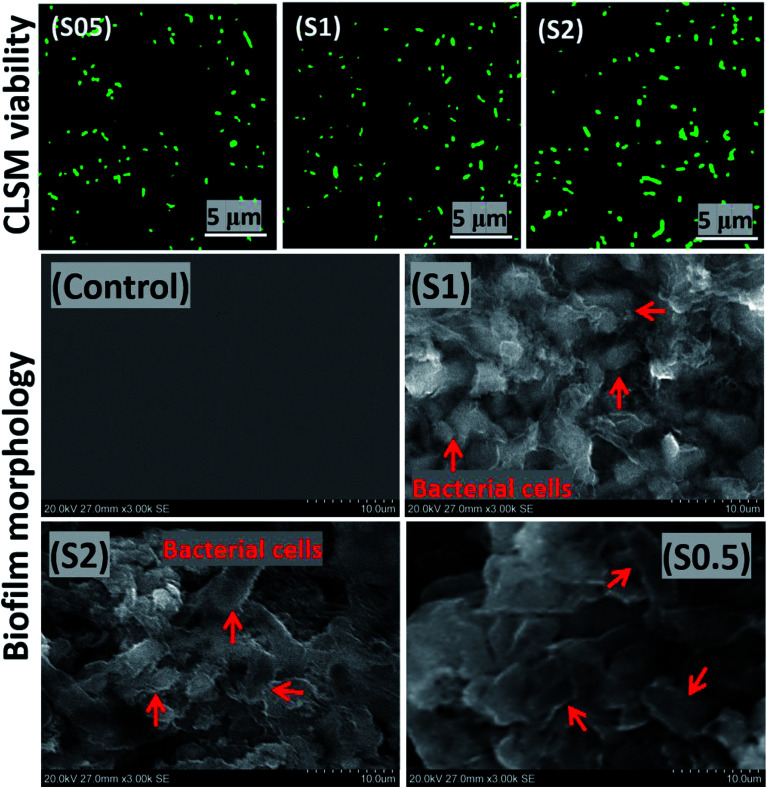
Scanning fluorescence micrographs showing *P. gingivalis* cellular clusters from adhering bacterial biofilms on transparent coverslips (upper row) and SEM micrographs showing *P. gingivalis* cellular clusters from adhering bacterial biofilms on stainless steel substrates (lower row) after 90 day incubation in ASCS media.

Artificial salivary culture suspension (ASCS) was the test media in this study containing varying concentrations of *P. gingivalis* seeds; 5 × 10^6^ CFU (S0.5), 10^7^ CFU (S1) and 2 × 10^7^ CFU (S2). The control system was a full uninoculated ASCS medium. The bacterial culture media were incubated for 90 days, and their inherent cellular concentration increased substantially to 1 × 10^7^ CFU in S0.5, 3.8 × 10^7^ CFU in S2 and 2 × 10^7^ CFU (S1), respectively, due to the formation of *P. gingivalis* biofilm. This is depicted in the viable bacterial cell clusters presented in Fig. 3 from the adhering biofilm growths on solid substrates. The isolated viable cells in these images are illuminated green. There may be no district changes in the observed fluorescence imaging, however, more cell counts were recorded for S2 compared to S1 and S0.5. The observed bacterial cells were close enough to the surface, so there was no need to view stack images within the biofilm depths. The presence of these bacterial biofilms revealed inherent cellular colonization, and this is consistent with the onset of dental periodontitis disease.^45^

### 3.2. Biofilm analyses by SEM and XPS

**Fig. 4:**
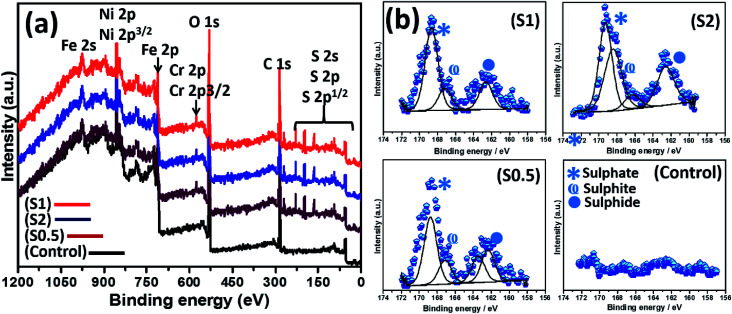
XPS wide-scan spectra (a) of *P. gingivalis* biofilms on stainless-steel substrates after 90 day incubation in ASCS media; (b) corresponding high-resolution valence peaks of S2p XPS spectra for S0.5–S2 and the experimental control.

The abundance of green-stained bacterial cells in Fig. 3 (upper row) depicts viability, and their visual SEM images were also recorded by means of SEM (lower row). The presented SEM micrographs show large bacterial colonies within adhering bacterial biofilms on the surfaces of steel substrates. The biofilms are densely packed cellular clusters fused within secreted extracellular polymeric substances (EPS) matrix that aid in cell–metal anchorage.^25^ The presented biofilm architecture reveals unique monospecies and aggregated colonies with randomized layered distributions.^45^ The distribution of bacterial cells within these biofilms between S1 and S0.5 is entirely randomized and also consistent with earlier reports in ref. 45–47. These biofilms are made of Gram-negative cellular colonies with short rod-shapes still capable of cell–cell quorum sensing within them. The XPS wide-scans spectra are presented in Fig. 4(a). The chemistry of adhering *P. gingivalis* biofilm on the stainless-steel substrates from XPS evidence are comparatively similar. The Fe, Ni and Cr contents are consistent with the base stainless-steel substrate while peaks of other elements could be linked with the bacterial biofilms. *P. gingivalis* is a biofilm-forming bacterium also known to release volatile sulfur compounds during its metabolic process.^22^ The presence of S2p peaks are related to these biofilm-bound volatile compounds; it should be noted that there are no S-related peaks on the control system. Fig. 4 also presents a collection of valency peaks of S2p spectra (b) of adhering biofilms on stainless steel substrates immersed in test ASCS media. Except for the control system, the shapes of sp^2^ valence peaks are similar with distinct triple peaks at 162 eV (sulphide), 167 eV (sulphite) and 169 eV (sulphate) due to these volatile compounds. Other authors have reported peaks corresponding to corrosion products from metal-based elements (*e.g.* FeS and MnS) in biofilm aggregates from other bacteria.^24^

### 3.3. Examining the extent of corrosion of stainless-steel substrates in ASCS media by SEM

**Fig. 5:**
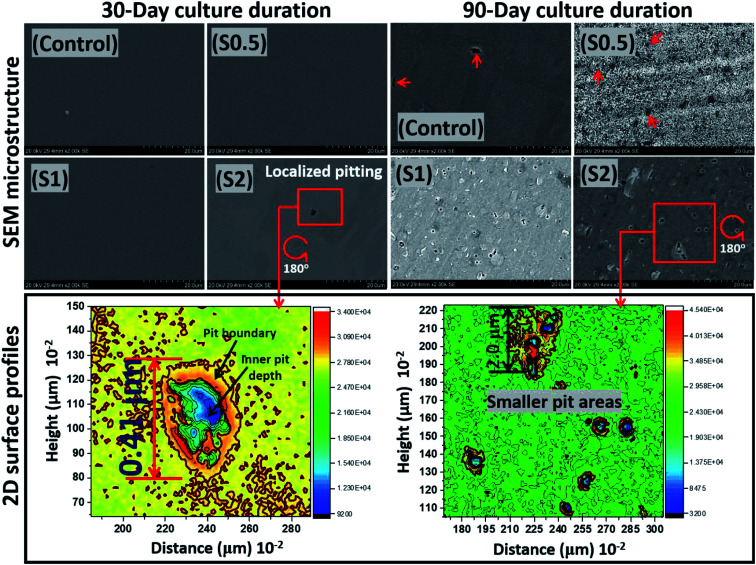
SEM micrographs and two-dimensional (2D) plane-view surface contour profiles showing the extent of corrosion of stainless-steel coupons after 30 and 90 day incubation periods within ASCS media. Localized pit area depths of 0.41 and less than 2 μm could be observed after 30 and 90 days, respectively, for S2. *P. gingivalis* cells, like any other bacterial cells, can penetrate metallic crevices when swimming towards food sources within the culture medium as they adsorb nutrients.^53^ MIC is initiated due products of the bacterial metabolic processes acting selectively at the surface pits and their neighbouring regions in such a way that corrosion rates are raised.

At the end of the 90 day incubation period, all stainless-steel substrates within the bioactive ASCS media were deactivated by immersing them in 2% glutaraldehyde in PBS fixation solution. This was closely followed by dehydration in anhydrous-ethanol solution before drying with N_2_ gas.^42^ These fixating and cleaning steps significantly removed adhering *P. gingivalis* colonies from the metallic surfaces before analyzing for evidences of surface pits using SEM imaging. As presented in Fig. 5, except for S2, there were no changes in the surface morphologies at the end of Day 30 due to the corrosion-resistant features within this metallic substrate, including its titanium-stabilized, metastable chromium phases and twins, stable-end orientations within its austenitic stainless-steel microstructure.^41^ For this duration of test, the rate of passivation of stainless steel by far exceeds its corrosion rate, even within a bacterial-inoculated media. This stainless-steel dental substrate is highly resistant to anodic dissolution; it is capable of forming martensite phases, if deformed. This was clearly consistent with the crystallographic orientation of the substrate in Fig. 1(c). These microstructural phases are known to resist corrosion,^41^ and there has been several reported studies in regarding these assertions, in both undeformed and deformed systems.^41,48–50^ The observed surface pits in the steel substrate incubated in the ASCS media containing 2 × 10^7^ CFU (*i.e.* S2) is suggestive of the consequence of biocorrosion in the presence of *P. gingivalis.* This rather tiny pitting episode could be a source of failure in dental implants fabricated from this medical-grade stainless steel (*e.g.* crowns). After an extended incubation period (90 days), the pits became significant on these substrates.

Between all substrates, less surface areas were significantly impacted by the corrosive effects of these *P. gingivalis* biofilms. The lower surface contour profiles also reveal the extent of pits from two-dimensional images on impacted surfaces. Localized pits were also profiled deeply through continuous points of unequal centered depressions initiated by the resident oral bacterium. The surface 2D contour profiles mapped wide depths of localized pitting patterns on steel surfaces exposed to S2 medium on both days. These areas were covered by the widest observable pits with less than 1 cm^2^ and rather uneven curvature, deeper at the blue spots relative to red due to MIC attack on steel. Measured pit lengths up to 0.41 and less than 2 μm were observed on steel at 30 and 90 day incubation periods, respectively, for S2. The comparative differences between the observed metal surfaces for both incubation periods could be linked with: (a) sufficient duration for corrosive impact after 90 days, and (b) maturity of growing *P. gingivalis* cells. The formation of pits, as observed within this study, could also have originated from *P. gingivalis* biofilm-led creation of corrosive concentration cells on these metal surfaces. Surface pits are a consequence of microbiologically induced corrosion (MIC), and the evolution of volatile sulphide compounds (*e.g.* H_2_S) also catalyzed electrochemical reactions capable of leading to the observed localized pitting episodes.^51–53^

### 3.4. Elemental contents released from corroded stainless-steel dental substrates

**Fig. 6:**
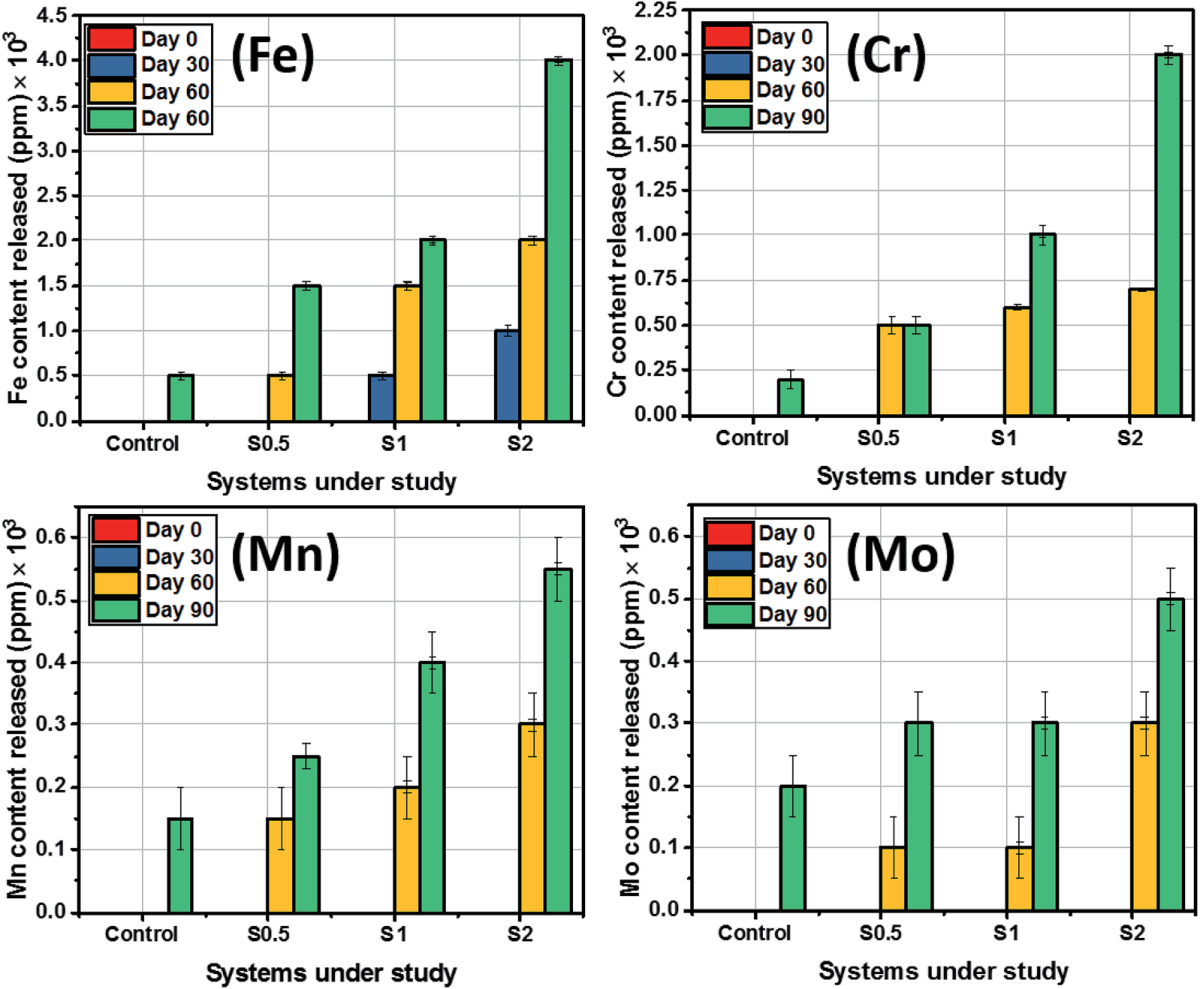
Amount of substrate-based elemental content released into ACS media from the stainless-steel dental substrate after 90 day incubation period.

The amount of substrate-based elemental contents released into the artificial salivary culture media per 1 square-centimeter surface area was also measured in ppm using ICP-MS. Before determining the concentrations of these major elements within these media, the metallic dental substrates were appropriately diluted with 1 M HCl in vortex mixer for 1 min. Stainless steel 321 is known for its supplementary Cr and Mn content, however, Fe still makes the balance as presented in Table 1. As expected, there were no substrate-based elemental release as ions immediately after immersion of the sterilized and pretreated dental substrates in the culture medium as presented in Fig. 6. However, at the end of Day 30 of incubation, the amounts of other elements released into the media were half that of Fe (500 ppm) from the same substrate. Fe content in all systems, including the control, appeared to be the most released from the substrates after corrosion. Higher amounts of ions from the steel were released upon steel dissolution at 90 day incubation period. It is worthy of note to mention that the mean levels of Mn and Mo eluted from steel were close while Mo contents between culture media with S0.5 and S1 were unchanged between Day 30 and 60. The amounts of Fe released ranged from 10 to 400 ppm in all test media for all incubation periods. Between the span of incubation period studied, the H_2_S levels ranged from 6.2 ppm (Day 30) to 8 ppm (Day 60) for ASCS media with the highest CFU (S2) while mercaptan levels stood between 5.6 to 7 ppm. The level for these sulphide products were lower for the culture medium with S0.5 or S1. Concentrations of H_2_S up to 5.5 and 5 ppm were recorded in Day 30 and 60, respectively, while 5.2 and 5.8 ppm mercaptan were also measured at the same period for S0.5. H_2_S levels stood at 6 and 7.5 ppm at Days 30 and 60 for S1 while lower amounts of mercaptan (6.4 and 6.8 ppm) were also measured between both durations. It is also worthy of note to mentioned that levels of sulphide products significantly reduced at Day 90 for all systems under study due to bacterial growth maturity. None of these volatile sulphide products were detected in the control medium. Elution of these volatile sulphide products was also accompanied by steady changes in media pH.

### 3.5. Correlating biocorrosion with weight-loss and electrochemical parameters within ASCS media

**Fig. 7:**
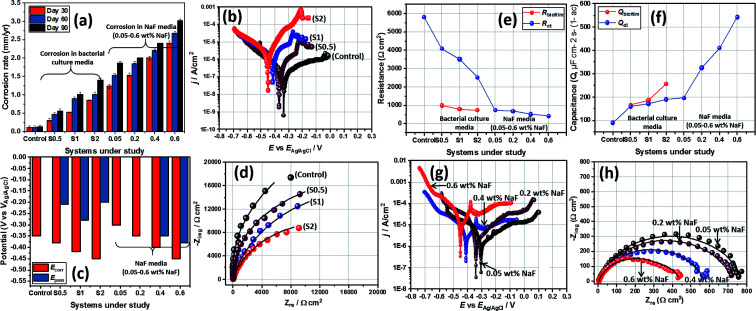
Electrochemical parameters: (a) corrosion rates of stainless-steel substrates immersed within ASCS media, with and without NaF after different incubation periods; (b) Tafel polarization curves and (c) their electrochemical parameters; (d) Nyquist curves of steel substrates after Day 90 within ASCS media; variation of (e) resistance and (f) capacitance parameters between biosystems under study. Tafel polarization (g) and Nyquist (h) curves for stainless-steel coupons immersed within ASCS media altered with different concentrations of NaF after a 90 day immersion period.

Significant anodic degradation in the forms of localized pitting has been observed for stainless-steel substrates 30 and 90 day incubation periods within ASCS media growing *P. gingivalis* cells. This was further investigated using weight loss and electrochemical techniques. The corrosion rate of each substrate between both incubation periods (Days 30 and 90) were computed from measured values of weight loss (stainless-steel metallic weight prior to and at the end of these incubation periods). These computed values of corrosion rate are presented in Fig. 7(a). At Day 30, the corrosion rates of steel substrates stood at 0.31, 0.52 and 0.85 mm year^−1^ for substrates incubated within culture media with S0.5, S1 and S2, respectively. At Day 90, the corrosion rate of steel increased to 0.56, 1.02 and 1.40 mm year^−1^ for steel substrates incubated within S0.5, S1 and S2, respectively, relative to the control (0.12 mm year^−1^ without the Gram-negative bacterial seeds). Higher corrosion rates were recorded for metallic substrates as a direct contribution of induced biocorrosion in the presence of *P. gingivalis* within the artificial salivary culture suspension. Steel corrosion rate increased with bacterial incubation periods for all metallic substrates, and in all systems, substrates incubated within ASCS media with double the CFU (S2) corroded more than S1 and half the CFU in S1 (*i.e.* S0.5). The observed corrosion of steel substrates must have also been due to MIC-led anodic dissolution incited by the products of bacterial metabolism.^24,25^

Results obtained from the weight loss technique was complemented with those from electrochemical technique. The first test involved polarizing these steel substrates by applying ±0.5 V at a 0.5 mV s^−1^ sweep rate, and in the end, changes in the magnitude of corrosion current density (*j*_corr_) were examined at Day 90 incubation period. Electrochemical potential values were estimated relative to the reference electrode, Ag/AgCl (sat. KCl) with each measurement collected at the end of 30 day incubation period. The polarization curves of stainless-steel substrates within ASCS media are depicted in Fig. 7(b). As expected of stainless steel, the shapes of anodic polarization curves reveal that stainless steel showed trans-passive behaviours toward −0.21, −0.28 and −0.20 V in S0.5, S1 and S2 media, respectively. However, values of corrosion current density (*j*_corr_) for these substrates increased in the order: control (0.1 μA cm^−2^) < S0.5 (2.0 μA cm^−2^) < S1 (5.0 μA cm^−2^) < S2 (11.2 μA cm^−2^); these changes are consistent with corrosion rate. Stainless-steel corroded more in ASCS media with S2 than S1 and S0.5, in the presence of *P. gingivalis*, and this is suggestive of accelerate steel MIC for double the CFU within the culture medium. The values of corrosion potential (*E*_corr_) for steel also stood at −0.38, −0.42, −0.45 and −0.35 V for S0.5, S1, S2 and control, respectively (Fig. 7(c)).

The extent of corrosion of steel substrates within ASCS media was also investigated with the aid of a noninvasive electrochemical impedance spectroscopic (EIS) technique. Its representative Nyquist spectra for stainless-steel coupons after 90 day incubation period within ASCS media are presented in Fig. 7(d). These impedance curves are single semi-circles with incomplete capacitive loops between real and imaginary axes. In this study, curves with wider diameters are consistent with more resistive metallic steel systems due to their corrosion resistance. In order to further probe the corrosion resistance of these metallic substrates, these impedance curves were fitted into suitable equivalent circuit model (*R*_soln_ (*Q*_biofilm_(*R*_biofilm_(*Q*_dl_(*R*_ct_))))). *R*_biofilm_*Q*_biofilm_ was introduced in order to account for the adhering bacterial biofilm on steel (Fig. 3). The values of electrical parameters collected from the curves in Fig. 7(d) are also presented within the ESI (Table S1†). *R*_soln_ values stood at 0.9, 2.0, 2.5 and 3.1 Ω cm^2^ for control, S0.5, S1 and S2, respectively. Increase in values of *R*_soln_ for bacterial-inoculated systems with higher CFU are contributions of bacterial metabolic processes within the ASCS media as volatile sulfur compounds are released.^23,54^ Reduced trend in *R*_biofilm_ and *R*_ct_ for inoculated systems relative to the control denotes MIC as the steel substrate slowly losses its corrosion resistance within the *P. gingivalis* culture.

Besides changes in *R*_ct_ (Fig. 7(e)), constant phase element (*Q*, *Y*_o_) values (both *Q*_biofilm_ and *Q*_dl_) were also monitored for all systems under study. As presented in Fig. 7(f), a continuous increment in *Q* was observed in the order: control > S0.5 > S1 > S2. It was important in order to probe the relationship between capacitance components (*Q*) and corrosion resistance since their introduction within the circuit model would account for inherent metal surface defects. These defects contributed to depressions and inherent nonuniform impedance spectral curvature due to distortions on the double-layers. The impedance of CPS is presented as *Z*_CPE_ = 1/*Y*_o_(*jω*)^*α*^; where 
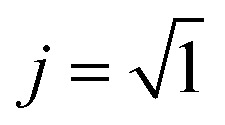
 and *ω* = 2π*f*. *ω*, *f* and *j* are magnitudes of angular frequency (measured in rad s^−1^), frequency (measured in Hz) and imaginary number, respectively. Magnitudes of *Y*_o_ as a quantity of *Q* describes inherent properties of adsorbed electroactive species while *α* is the phase shift whose values are within −1 and 1.^25,41,54^ Nyquist curves were recorded at *E*_oc_; potential-time *E*_oc_ evolution plots for selected test systems within the 90 day incubation duration are represented in Fig. S1(a) and (b).† More negative potentials were observed for steel substrates in more corrosive media, at higher CFU and NaF additive concentrations.

### 3.6. Examining the extent of corrosion in NaF modified artificial saliva solutions

**Fig. 8:**
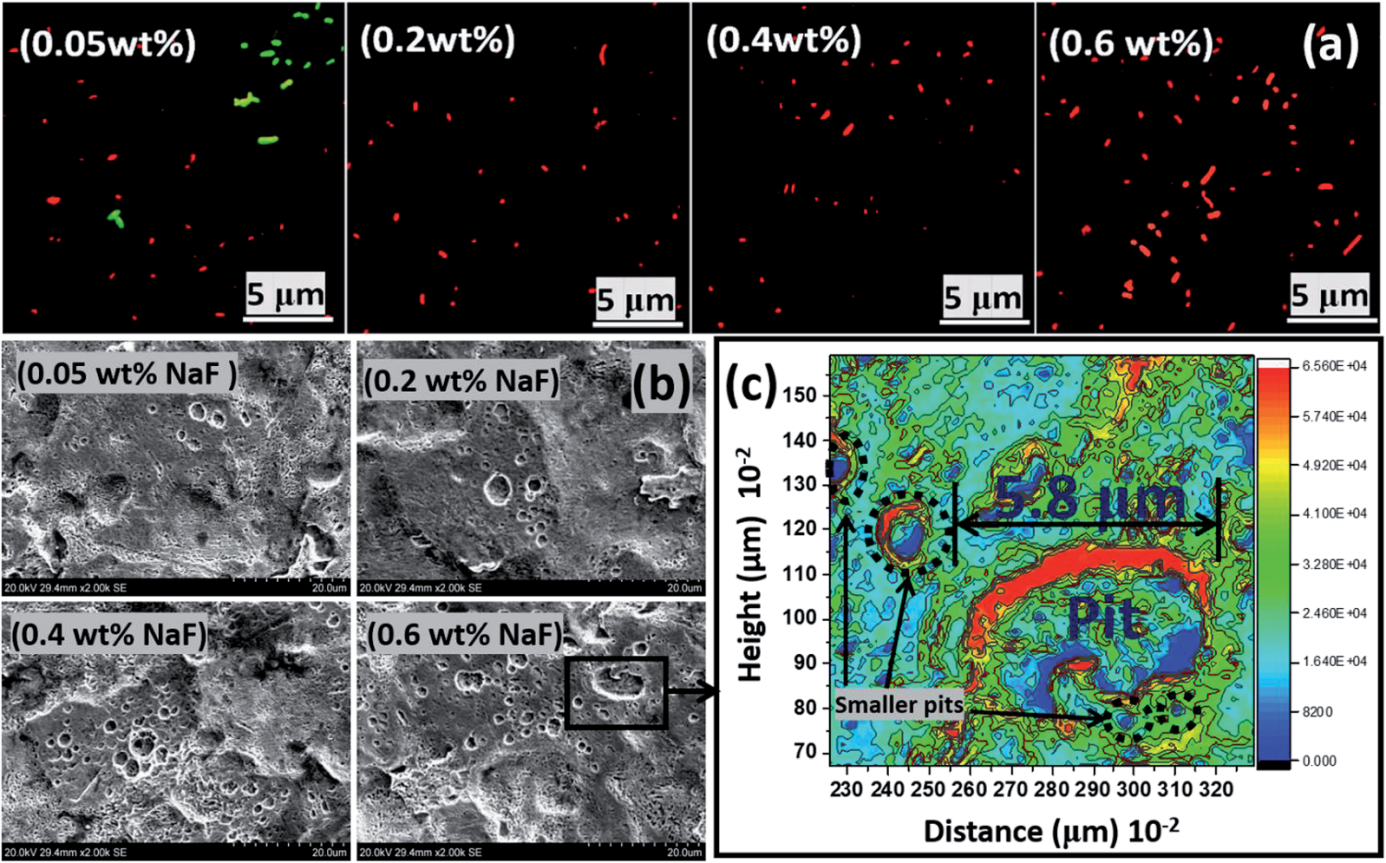
(a) Scanning fluorescence micrographs showing viable and dead *P. gingivalis* bacterial cells after 90 day incubation within NaF altered ASCS media. All steel coupons were incubated within bacterial culture with the highest CFU (S2). (b) SEM micrographs showing the extent of pitting corrosion of stainless-steel coupons after 90 day incubation period within ASCS media altered with different concentrations of NaF. (c) A surface contour profile mapping localized pitting patterns on steel surface in 0.6 wt% NaF altered ASCS media after 90 day immersion period upon the creation of electrochemical cells on the surface of the metallic substrate. The SEM micrograph for the control sample in NaF solution alone is presented within the ESI (Fig. S1(c)).†

Table 3 presents summaries of results from a few comparative studies showing the biocorrosion behaviours of different medical-grade metallic substrates in salivary culture media inoculated with *P. gingivalis* seeds as reported within this study and those reported in literature. Beyond the artificial salivary culture suspension, the corrosion behaviour of stainless-steel substrates in NaF altered culture media was also investigated. This was a simulated high fluoride (NaF) enriched artificial salivary media designed to examined external influences on steel corrosion. NaF was added as a principal component of many fluoride-containing toothpaste and mouthwashes, and also noted for its antibacterial activities.^31^ Since NaF is also known to further reduce the pH of oral media to acidic values, it was necessary to further investigate changes in corrosion resistance of the stainless-steel crown material in the presence of these fluoride ions.^31^ Before the corrosion test, the extent bacterial survival at varying chosen concentrations (0.05–0.6 wt%) of NaF within the media was examined while monitoring the corrosion of stainless-steel dental substrates. Fig. 8(a) depicts scanning fluorescence micrographs showing significant dead *P. gingivalis* bacterial cells after 90 day incubation within ASCS media altered with NaF. Results from the weight loss technique reveal significant corrosion rate for each stainless steel dental substrate between the incubation periods under study as represented in Fig. 7(a). At Day 30, the corrosion rate of steel stood at 1.23, 1.54, 2.00 and 2.41 mm year^−1^ for steel substrates incubated within 0.05, 0.2, 0.4 and 0.6 wt% NaF, respectively. At Day 60 and 90, the corrosion rate of steel stood at 1.53 and 1.86; 1.85 and 2.00; 2.20 and 2.30; and 2.68 and 3.01 mm year^−1^ for steel substrates incubated within 0.05, 0.2, 0.4 and 0.6 wt% NaF, respectively. Higher corrosion rates are recorded for stainless-steel coupons as a consequence of intense chloride-induced corrosion within the saline test media. Steel corrosion rate increased with immersion time for all substrates as well as with increased NaF content and not biocorrosion (since there were no viable bacterial cells).

**Table 3:** Comparison between the biocorrosion behaviours of different medical-grade metallic substrates in salivary culture media inoculated with *P. gingivalis* seed as reported within this study and those reported in literature^a^

S/No	Application of metallic substrate	Type of oral bacterium/culture conditions	Initial bacterial concentration	Key findings	Ref.
1.	Stainless-steel (AISI 321) dental crown	*P. gingivalis* (ATCC 33277)/incubated at 38 °C for 30–90 days under anaerobic conditions in artificial saliva culture suspension (pre-culture in BHI broth) with NaF alteration	10^7^ CFU mL^−1^	Significant anodic degradation in the forms of localized pitting were observed as consequences of processes leading to bacterial metabolism, biofilm growth and MIC. Surface pitting was severe in the presence of NaF within the salivary culture media.	This study
2.	Pure titanium implant disk	*P. gingivalis* (ATCC 33277)/incubated aerobically at 37 °C for 3–14 days in BHI broth	10^7^ CFU mL^−1^	Sulphide products were produced by adhering *P. gingivalis* biofilms on titanium disks; however, this did not result in corrosion.	29
3.	Stainless steel orthodontic archwires	No bacterial culture/acidic NaF-containing artificial saliva	—	The corrosion of orthodontic archwires was significant in the presence of NaF and prolonged exposure period; this also led to increase surface roughness due to formation of fluoride complexes.	31
4.	Stainless steel orthodontic brackets coated with TiO_2_ mixed Ag	*P. gingivalis* (ATCC 33277)/incubated at 37 °C for 1 day under anaerobic conditions in lysogeny broth agar	5 × 10^8^ CFU mL^−1^	The TiO_2_–Ag film on the steel brackets prevented against bacterial surface adhesion and biofilm formation due to its antibacterial properties and resistance toward plaque accumulation.	35
5.	Orthodontic stainless-steel brackets	*P. gingivalis* (DSM 20709)/incubated at 37 °C for 3 days under anaerobic conditions in saliva solutions of healthy adults	10^8^ CFU mL^−1^	The salivary pellicle facilitated the adhesion and formation of *P. gingivalis* biofilms on orthodontic brackets.	36
6.	SLA titanium dental implant surfaces	*P. gingivalis* (ATCC 33277)/incubated at 37 °C for 7 days under anaerobic conditions in BHI culture	10^8^ CFU mL^−1^	Bacterial cells significantly covered the metallic titanium surfaces in turn weakening their surface properties. This led to reduced protective properties of TiO_2_ film, resulting in biocorrosion. These corroded surfaces also exhibited lower osteocompatibility and reduced adhesion of MC3T3-E1 cells.	63
7.	304 Cu-bearing austenitic antibacterial orthodontic stainless steel	*P. gingivalis* (ATCC 33277)/incubated at 37 °C for 3 days under anaerobic conditions in BHI–S blood agar	10^9^ CFU mL^−1^	The metallic substrate showed no significant antibacterial activity toward *P. gingivalis* growth; there were also no observable cytotoxicity toward osteosarcoma MG-63 cells and oral epithelioma KB cells.	64
8.	Ti–Cu sintered alloys	*P. gingivalis* (ATCC 33277)/incubated at 37 °C for 3 days under anaerobic conditions in BHI agar	1.5 × 10^9^ CFU mL^−1^	*P. gingivalis* cells growth significantly reduced with Cu content (with 50% after 24 h alloy incubation) but increased culture duration.	65
9.	Pac-525 coated titanium implant substrate	*P. gingivalis* (ATCC 33277)/incubated aerobically at 37 °C for 2 days in BHI broth	10^8^ CFU mL^−1^	Pac-525 inhibited *P. gingivalis* biofilm adhesion, biofilm growth and cellular growth on titanium dental implant surface.	66

a ATCC: American Type Culture Collection.

As presented in the Tafel curves (Fig. 7(g)), there are also significant changes in values of *E*_pass_ and *E*_corr_ for steel substrates in NaF (Fig. 7(d)). Except for 0.05 and 0.2 wt% NaF, other substrates demonstrated electrochemical behaviors that were consistent with anodic trans-passivation character. Values of *j*_corr_ increase with NaF concentration in the order: 0.6 wt% NaF (90.1 μA cm^−2^) > 0.4 wt% NaF (75.5 μA cm^−2^) > 0.2 wt% NaF (55.0 μA cm^−2^) > 0.05 wt% NaF (22.0 μA cm^−2^). This trend is suggestive of increased corrosion rate at higher fluoride ionic concentrations. Other electrical parameters related to the Nyquist spectra (Fig. 7(h)) for stainless-steel dental substrates within the NaF modified ASCS media are also presented in the table. Compared to the impedance curves for metallic substrates without NaF (Fig. 7(c)), those in the presence of NaF depict semi-circles with complete capacitive loops. Metallic systems with higher corrosion resistance have wider Nyquist curve diameters. The impedance curves were fitted into suitable equivalent circuit model (*R*_soln_ (*Q*_dl_(*R*_ct_))). The values of electrical parameters (Fig. 7(e) and (f)) collected from the curves are presented in Table S1.† While *Q*_dl_ values increased with NaF concentrations, reduced magnitudes of *R*_ct_ were observed for dental substrates within the media; 732.2, 685.1, 515.9 and 423.0 Ω cm^2^ for 0.05, 0.2, 0.4, 0.6 wt% NaF, respectively. Since the impedance curves within this study were collected at *E*_oc_, the corresponding *E*_oc_ plots for all tested samples within the 90 day incubation duration are presented within the ESI.† More negative values of potential were recorded for steel within more corrosive systems relative to the control. Similar trends were observed in both potential-time evolution curves both with and without the NaF additives in the culture media. In both cases, corrosion of steel was significantly affected by the incubation process. The trend of electrochemical data is consistent with those of the weight loss technique and confirms that stainless-steel corrosion increased with NaF concentrations.

To further probe the electrochemical results, the morphologies of impacted metallic surfaces were also examined for corrosion-related changes. SEM micrographs showing the extent of corrosion of stainless-steel dental substrates after 90 day incubation period within ASCS media altered with different concentrations of NaF are presented in Fig. 8. Significantly surface pits were observed as a result of fluoride-induced corrosion with increasing NaF concentrations. These metallic substrates had the tendency to dissolve within this acidic media, hence the increase number of pit areas at higher NaF levels. The surface 2D contour profile mapped wide depths of localized pitting patterns on steel surface exposed to 0.6 wt% NaF due to saline corrosion at specific metallic grains and the high chloride content of the media. These localized pits were also profiled deeply through continuous points of unequal centered depressions while the depths of contoured lines on corroded surface show patterns consistent with SEM image. The observed pit sites in the presence of NaF appeared to show extreme degradation on the metal surfaces from depassivation of localized anodic portions caused by the creation of galvanic cells with nearby wider cathodic sites due to unrestricted attack by fluoride ions.

Austenitic stainless steels, like the medical-grade material utilized in this study, are highly resistant to corrosion due to their metastable chromium enriched phases. When stressed upon persistent application of loads (*e.g.* during mastication of food particles of varying hardness and sizes), these materials eventually undergo martensitic transformation, with the maximum masticatory force reaching values of 500–700 N in some adult humans. So, it became pertinent to investigate the susceptibility of this medical-grade metastable austenitic stainless-steel dental grade (AISI 321) substrate to localized pitting corrosion initiated by the acidic NaF media. Like most austenitic steels, this dental substrate was stabilized with titanium (0.36 wt% Ti in Table 1) in order to avert the depletion of chromium around the grain boundaries (*i.e.* intergranular corrosion). This could have occurred at 500–800 °C,^55–57^ but since there were no high-temperature treatments in this study, there were also no observed surface evidences of intergranular corrosion, with the grain-boundary chromium contents remaining intact. However, the stainless-steel dental substrates still corroded with localized pitting patterns (see Fig. 8(b)) and the surface contour profile (c) also revealed the extent of fluoride-induced pitting on steel after 90 day immersion in 0.6 wt% NaF media. Fig. S1(c)† depicts SEM micrographs of the control sample within undoped NaF solution. Compared to SEM of the test samples, there are less presence of pits at the end of the culture duration; this is suggestive of biocorrosion in the presence of the growing *P. gingivalis* bacterial biofilms. The corrosion rate of steel was high even in the presence of adhering fluoride complexes formed on the surface of metallic substrates at pH ≈ 1 in 0.6 wt% NaF.^31^ These fluoride complexes were removed using 2% glutaraldehyde in PBS fixation and repeated sonication using anhydrous ethanol in order to recorded high-resolution images with clarity. The XRD patterns of these fluoride complex thin films deposited on impacted stainless-steel substrates are presented in Fig. S1(d).† As expected, these complexes composed of a mixture of simple metallic fluoride (FeF_2_, FeF_3_, CrF_3_ and NiF_2_) and few ferrous oxides (Fe_2_O_3_ and Fe_3_O_4_).^58–62^ Mouth rinsing products with fluoride ions up to 250–10 000 mg L^−1^ concentrations promote the formation of these metallic fluorides that eventually lead to remineralization between pH 3.5–7. This is slowly proceeded by corrosion of dental implants within this acidic NaF environment and at high concentration and pH. The prevalence of fluoride ions also further degrades the protective oxide films formed on the dental implants upon contact with the acidic electrolyte. Most of these oxide layers may be ferrous oxides as depicted in Fig. S1(d).†

## 4. Conclusions

In this study, *in vitro* corrosion behaviour of medical-grade stainless-steel dental substrates was investigated during *Porphyromonas gingivalis* biofilm growth in artificial saliva culture suspension. Bacterial growth increased significantly within the incubation period under study. This was also accompanied by metabolic processes leading to steady elution of mercaptan and hydrogen sulphide products that also peaked with growth maturity. XPS evidence also revealed S2p valency peaks consistent with sulphur-based volatile compounds from adhering biofilms on stainless steel substrates due to these volatile sulphur compounds. Adhering *P. gingivalis* biofilms on the surfaces of stainless-steel dental substrates resulted in significant anodic degradation in the forms of localized pitting within the artificial salivary culture suspension media. Elemental release of substrate-based components (as ions) were also determined by ICP-MS technique following the corrosion of steel substrates. Corrosion rate increased at higher CFU as well as with bacterial culture duration for all substrates due to biocorrosion. The presence of residual fluorinated ions within the ASCS media also contributed to increased rates of anodic dissolution, hence corrosion, after 90 days. The acidity of these fluoride media (0.05–0.6 wt% NaF) led to loss of chromium depletion at the grain boundaries, thereby resulting in significant metal-surface pitting. Corrosion investigations in acidic oral conditions containing fluoride ions was necessary in order to depict fluoride-containing oral environments during mouthwashes and toothpastes to avoid dental cavity and surface sensitivity. Stainless steel is used in fabricating dental crowns and other forms of implants within the oral cavity. However, these metallic substrates eventually degrade due to surface pitting and inherent cracks being consequences of biocorrosion and mechanical stresses. This study features an attempt to understand the corrosion patterns of a stainless-steel dental substrate in a typical oral media growing a biofilm-forming Gram-negative oral bacterium.

## Declaration

Authors wish to declare that none of the experiments within this study was conducted on humans neither was the use of human tissue samples utilized. This study features the degradation and biocorrosion patterns of a stainless-steel dental substrate in simulated/artificial saliva with composition presented in Table 2.

## Conflicts of interest

There are no conflicts of interest.

## Supplementary Material

RA-010-D0RA05500J-s001

## References

[cit1] Kahyarian A., Brown B., Nesic S. (2017). Corros. Sci..

[cit2] Yule L. C., Shkirskiy V., Aarons J., West G., Shollock B. A., Bentley C. L., Unwin P. R. (2020). Electrochim. Acta.

[cit3] Humphry W. P. (1950). Dent. Surv..

[cit4] Sharaf A. A., Farsi N. M. (2004). J. Dent..

[cit5] Salama F. S., Alowyyed I. S. (2001). Dent. News.

[cit6] Salama F. S. (1996). Cairo Dent. J..

[cit7] Durr D. P., Ashrafi M. H., Duncan W. K. (1982). J. Dent. Child..

[cit8] Webber D. L. (1974). J. Dent. Child..

[cit9] Checchio L. M., Gaskill W. F., Carrel R. (1983). J. Dent. Child..

[cit10] Randall R. C., Vrijhoef M. M., Wilson N. H. (2000). J. Am. Dent. Assoc., JADA.

[cit11] Bin A. W. M., El-Shehaby F. A., El-Dokky N. A., Reda A. R. (2012). J. Indian Soc. Pedod. Prev. Dent..

[cit12] Ludwig K. H., Fontana M., Vinson L. A., Platt J. A., Dean J. A. (2020). J. Am. Dent. Assoc.,JADA.

[cit13] Srivastava V. K. , Various Designs of prefabricated crown in pediatric dentistry, in Modern Pediatric Dentistry, JP Medical Ltd, 2011, ch. 24,pp. 1–390

[cit14] Fenner N., Hämmerle C. H. F., Sailer I., Jung R. E. (2016). Clin. Oral Implants Res..

[cit15] Goodacre C. J., Bernal G., Rungcharassaeng K., Kan J. Y. K. (2003). J. Prosthet. Dent..

[cit16] Ribeiro C. G., Maia M. L. C., Scherrer S. S., Cardoso A. C., Wiskott H. W. A. (2011). J. Appl. Oral Sci..

[cit17] Shemtov-Yona K., Rittel D., Levin L., Machtei E. E. (2014). Clin. Oral Implants Res..

[cit18] Hardt C. R., Grondahl K., Lekholm U., Wennstrom J. L. (2002). Clin. Oral Implants Res..

[cit19] Brocard D., Barthet P., Baysse E., Duffort J. F., Eller P., Justumus P. (2000). Int. J. Oral Maxillofac. Implants.

[cit20] Rodrigues D., Valderrama P., Wilson T., Palmer K., Thomas A., Sridhar S. (2013). Materials.

[cit21] de Waal Y. C., Eijsbouts H. V., Winkel E. G., van Winkelhoff A. J. (2016). J. Periodontol..

[cit22] Persson S., Claesson R., Carlsson J. (1989). Oral Microbiol Immunol.

[cit23] Hu Y., Wu G., Li R., Xiao L., Zhan X. (2020). Water Res..

[cit24] Eduok U., Ohaeri E., Szpunar J. (2019). Mater. Sci. Eng., C.

[cit25] Eduok U., Faye O., Szpunar J. (2018). Eng. Failure Anal..

[cit26] Scarano A., Di Domizio P., Petrone G., Iezzi G., Piattelli A. (2000). J. Oral Implantol..

[cit27] Tseng C. C., Chen Y. H. M., Pang I. C., Weber H. P. (2005). Int. J. Oral Maxillofac. Implants.

[cit28] Xu W., Zhou W., Wang H., Liang S. (2020). Adv. Protein Chem. Struct. Biol..

[cit29] Harada R., Kokubu E., Kinoshita H., Yoshinari M., Ishihar K., Kawadaa E., Takemoto S. (2018). Dent. Mater. J..

[cit30] Persson G. R., Renvert S. (2014). Clin. Implant Dent. Relat. Res..

[cit31] Pulikkottil V. J., Chidambaram S., Bejoy P. U., Femin P. K., Paul P., Rishad M. (2016). J. Pharm. BioAllied Sci..

[cit32] Laurent F., Grosgogeat B., Reclaru L., Dalard F., Lissac M. (2001). Biomaterials.

[cit33] Hossain M. I., Mizan F. R., Ashrafudoulla M., Nahar S., Joo H. J., Jahid I. K., Park S. K., Kim K. S., Ha S. D. (2020). LWT–Food Sci. Technol..

[cit34] Trolic I. M., Serdarevic N. L., Todoric Z., Budimir A., Spalj S., Curkovic H. O. (2019). Surf. Coat. Technol..

[cit35] Fatani E. J., Almutairi H. H., Alharbi A. O., Alnakhli Y. O., Divakar D. D., Muzaheed D. D., Alkheraif A. A., Khan A. A. (2017). Microb. Pathog..

[cit36] Papaioannou W., Panagopoulos A., Koletsi-Kounari H., Kontou E., Makou M. (2012). Int. J. Dent..

[cit37] Yoshida T., Aoki H., Yoshida K., Irie N., Murayama D. (2009). J. Jpn. Soc. Dent. Prod..

[cit38] Di G. M., Traini T., Sinjari B., Nostro A., Caputi S., Cellini L. (2016). Clin. Oral Implants Res..

[cit39] Egawa M., Miura T., Kato T., Saito A., Yoshinari M. (2013). Dent. Mater. J..

[cit40] Noumbissi S., Scarano A., Gupta S. (2019). Materials.

[cit41] Tiamiyu A. A., Eduok U., Odeshi A. G., Szpunar J. A. (2019). Mater. Sci. Eng., A.

[cit42] Liu H., Gu T., Zhang G., Liu H., Cheng Y. F. (2018). Corros. Sci..

[cit43] Bjorklund M., Ouwehand A. C., Forssten S. D. (2011). Curr. Microbiol..

[cit44] de la Cruz A. B., Boahene P., Vedachalam S., Dalaia A. K., Adjay J. (2020). Fuel.

[cit45] Rogers S., Honm K., Mang T. S. (2018). Photodiagn. Photodyn. Ther..

[cit46] Standard Practice for Preparing, Cleaning, and Evaluating Corrosion Test Specimens, ASTM G1-03, 2001. (http://www.astm.org/Standards/G1.htm)

[cit47] Yamada M., Ikegami A., Kuramitsu H. K. (2005). FEMS Microbiol. Lett..

[cit48] Chen A. Y., Hu W. F., Wang D., Zhu Y. K., Wang P., Yang J. H., Wang X. Y., Gu J. F., Lu J. (2017). Scr. Mater..

[cit49] Kumar B. R., Singh R., Mahato B., De P. K., Bandyopadhyay N. R., Bhattacharya D. K. (2005). Mater. Charact..

[cit50] Phadnis S. V., Satpati A. K., Muthe K. P., Vyas J. C., Sundaresan R. I. (2003). Corros. Sci..

[cit51] Castaneda H., Benetton X. D. (2008). Corros. Sci..

[cit52] Sahrani F. K., Ibrahim Z., Yahya A., Aziz M. (2008). Sains Malays..

[cit53] Revie R. W. , Uhlig's Corrosion Handbook, John Wiley & Sons, Inc., 2nd edn, 2000, pp. 45–67

[cit54] Eduok U., Khaled M., Khalil A., Suleiman R., El Ali B. (2016). RSC Adv..

[cit55] Matula M., Hyspecka L., Svodoba M., Vodarek V., Dagbert C., Galland J., Stonawska Z., Tuma L. (2001). Mater. Charact..

[cit56] Kim S. B., Piak K. W., Kim Y. G. (1998). Mater. Sci. Eng., A.

[cit57] Hong H. U., Rho B. S., Nam S. W. (2001). Mater. Sci. Eng., A.

[cit58] Schwind M., Källqvist J., Nilsson J. O., Agren J., Andren H. O. (2000). Acta Mater..

[cit59] Prakash R., Mishra A. K., Roth A., Kubel C., Scherer T., Ghafari M., Hahn H., Fichtner M. (2010). J. Mater. Chem..

[cit60] Sanghvi S., Pereira N., Halajko A., Amatucci G. G. (2014). RSC Adv..

[cit61] Zhang H., Zhou Y. N., Sun Q., Fu Z. W. (2008). Solid State Sci..

[cit62] Rougier A., Makirnura Y., Penin N., Darok X., Tarascon J. M. (2006). J. Condens. Mater..

[cit63] Xu L., Yu X. Y., Chen W. Q., Zhang S. M., Qiu J. (2020). RSC Adv..

[cit64] Zhang D., Ren L., Zhang Y., Xue N., Yang K., Zhong M. (2013). Colloids Surf., B.

[cit65] Bai B., Zhang E., Liu J., Zhu J. (2016). Dent. Mater. J..

[cit66] Li Y., Wang X. J., Wang L. N., Ying X. X., Ren X., Liu H. Y., Xu L., Ma G. W. (2015). BioMed. Res. Int..

