# Supplementary material for: In vitro corrosion studies of stainless-steel dental substrates during Porphyromonas gingivalis biofilm growth in artificial saliva solutions: providing insights into the role of resident oral bacterium
Source: RSC Adv. 2020 Aug 24;10(52):31280–94. doi: 10.1039/d0ra05500j (PMC9056404; doi:10.1039/d0ra05500j)
Supplement: RA-010-D0RA05500J-s001 [file RA-010-D0RA05500J-s001.pdf]

Electronic Supplementary Material (ESI) for RSC Advances.
This journal is © The Royal Society of Chemistry 2020

In vitro corrosion studies of stainless-steel dental substrates during *Porphyromonas gingivalis* biofilm growth in artificial saliva solutions: Providing insights into the role of resident oral bacterium

Ubong Eduok*, Jerzy Szpunar

Department of Mechanical Engineering, College of Engineering, University of Saskatchewan, 57 Campus Drive, Saskatoon, S7N 5A9, Saskatchewan, Canada

SUPPORTING INFORMATION

Table S1. Electrical parameters for stainless steel substrates cultured within ASCS media, with and without NaF alteration, for 90-day incubation period.

System under study		R_{soln} (Ω cm^2)	R_{bioilm} (Ω cm^2)	$Q_{biofilm}$ (μF cm^{-2} s$^{-(1-\alpha c)}$)	R_{ct} (Ω cm^2)	Q_{dl} (μF cm^{-2} s$^{-(1-\alpha c)}$)	χ^2×10^{-4}	E_{corr} (V vs Ag/AgCl)	E_{pass} (V vs Ag/AgCl)	j_{corr} (μA/cm^2)	β_a (mV/dec)	β_c (mV/dec)
In *P. gingivalis* culture media	Control	0.9	–	–	5801.9	90.5	9.5	-0.35	–	0.1	63.5	124.9
	S0.5	2.0	990.1	166.1	4084.8	159.7	5.1	-0.38	-0.21	2.0	72.2	94.5
	S1	2.5	794.8	185.8	3508.6	171.0	7.5	-0.42	-0.28	5.0	87.4	147.7
	S2	3.1	731.0	257.7	2527.8	190.5	1.8	-0.45	-0.20	11.2	68.1	119.2
P. gingivalis in NaF altered media	0.05 wt.% NaF	3.2	–	–	732.2	196.7	5.2	-0.30	–	22.0	56.9	120.3
	0.2 wt.% NaF	4.2	–	–	685.1	325.4	4.6	-0.35	–	55.0	94.1	110.1
	0.4 wt.% NaF	4.0	–	–	515.9	410.0	7.5	-0.40	-0.35	75.5	45.8	145.5
	0.6 wt.% NaF	9.9	–	–	423.0	541.9	9.3	-0.45	-0.38	90.1	78.4	90.4

In order to further probe the corrosion resistance of stainless steel substates, impedance spectra of metallic coupons incubated within bacterial inoculated system were fitted into this equivalent circuit model [R_{soln} ($Q_{biofilm}$(R_{bioilm}(Q_{dl}(R_{ct}))))] while those in the control and NaF media were fitted in [R_{soln}(Q_{dl}(R_{ct}))] model. Goodness of Fit is represented as χ^2; values of α within this work are close to unity. Corrosion rate was not computed at Day 1 since there were no observed surface changes or corrosion episodes on the surfaces of polished stainless-steel dental substrates.

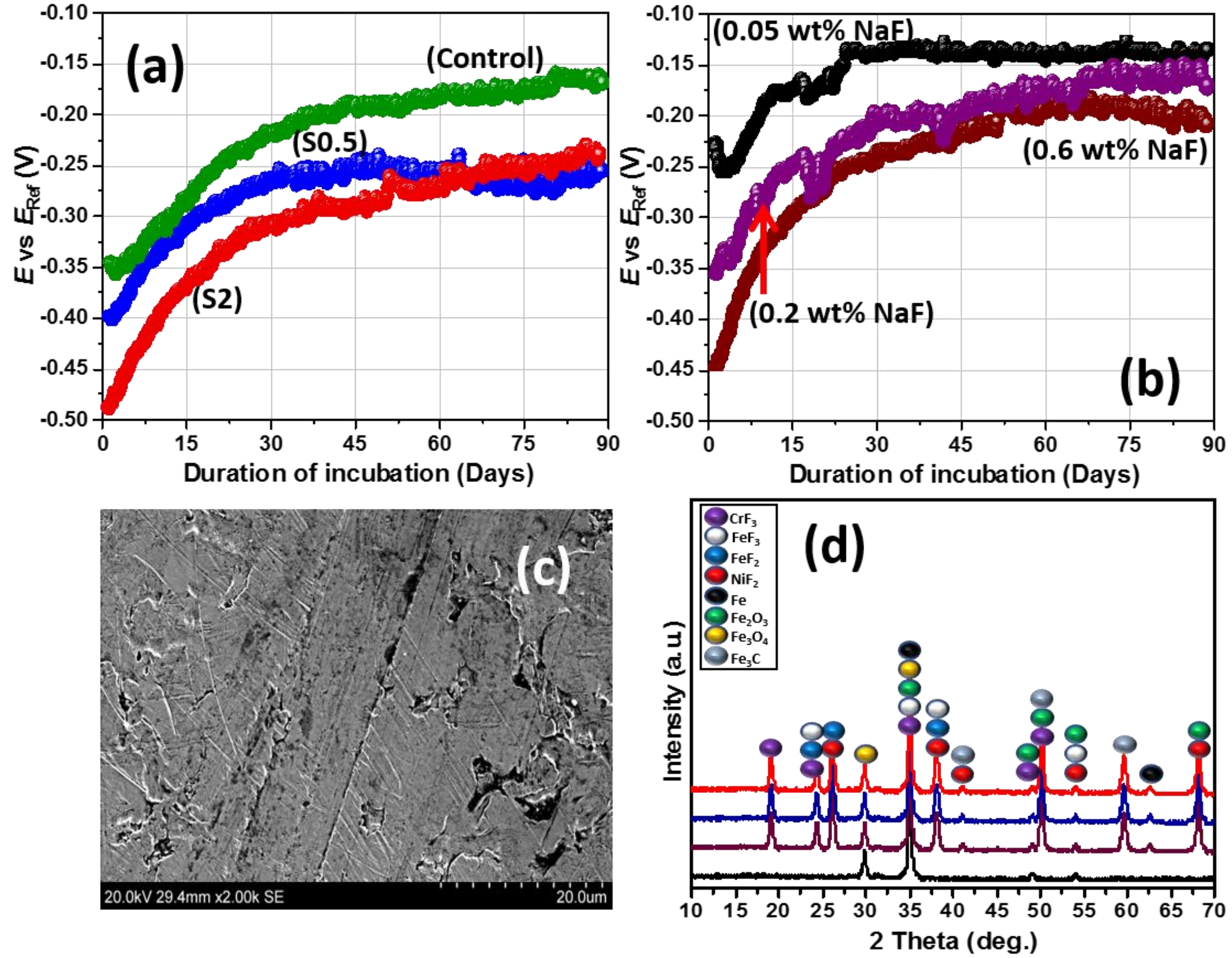

Figure S1. Potential-time E_{oc} evolution plots for selected test systems within the 90-day incubation (a) without and (b) with NaF additives. (c) SEM micrographs of the control sample within undoped NaF solution. Compared to SEM of the test samples, there are less presence of pits at the end of the culture duration; this is suggestive of biocorrosion in the presence of the growing *P. gingivalis* bacterial biofilms. (d) X-ray diffraction (XRD) patterns of fluoride complex thin films deposited on stainless steel substrates from NaF altered ASCS media. Spectra were recorded with the aid of a Bruker D8 Discover XRD Diffractometer with Cr─Kα radiation.
